# Employers’ Perception and Practice of Workplace Violence Prevention at Healthcare Facilities Questionnaire: A Confirmatory Factor Analysis

**DOI:** 10.21315/mjms2023.30.5.16

**Published:** 2023-10-30

**Authors:** Mohd Nizam Mohamad Yazid, Nik Rosmawati Nik Husain, Aziah Daud, Yelmizaitun Osman, Normazura Mustapa, Azlihanis Abdul Hadi

**Affiliations:** 1 Department of Community Medicine, School of Medical Sciences, Universiti Sains Malaysia, Kelantan, Malaysia; 2 Kelantan State Health of Department, Kelantan, Malaysia; 3 Melaka State Health of Department, Melaka International Trade Centre, Melaka, Malaysia; 4 Ministry of Health Malaysia, Federal Government Administrative Centre, Putrajaya, Malaysia

**Keywords:** workplace violence prevention, perception, practice, employer, questionnaire validation

## Abstract

**Background:**

Workplace violence prevention initiatives are undeniably lacking in healthcare facilities. The aim of this study was to validate a newly developed questionnaire and assess employers’ perceptions and practices towards workplace violence prevention at healthcare facilities.

**Methods:**

A cross-sectional study was conducted from October 2021 to November 2021 by recruiting 333 employers at healthcare facilities in Kelantan, Malaysia. The original draft of the Malay version of the questionnaire comprised 62 items constructed under two domains (perception and practice). A confirmatory factor analysis was conducted to evaluate construct validity and internal consistency using R software.

**Results:**

The final model for the perception and practice domain of the questionnaire consisted of 13 factors and 56 items. The factor loadings for all items were above 0.6. The fit indices used for confirmatory factor analysis in the final model were as follows: *χ*^2^ = 2092.6 (*P* < 0.001), standardised root mean squared residual (SRMR) = 0.053, root mean square error of approximation (RMSEA) = 0.042, comparative fit index (CFI) = 0.928 and Tucker Lewis index (TLI) = 0.920. The construct reliability for all factors was reliable, with Raykov’s rho coefficients above 0.70.

**Conclusion:**

The newly developed questionnaire demonstrated excellent psychometric properties and adequate validity and reliability, confirming that this instrument is reliable and valuable for evaluating employers’ perceptions and practices towards workplace violence prevention at healthcare facilities.

## Introduction

Working in healthcare sectors requires that healthcare workers (HCWs) be fully focused, committed and self-cautious. HCWs are exposed to violence in the workplace but they are still expected to provide the best healthcare services. Healthcare facilities are unavoidably prone to workplace violence (WPV), despite the availability of guidelines and training modules to prevent its occurrence. WPV occurs when workers are abused, threatened or attacked under conditions connected with their work, including their commutes to and from work, and are exposed threats to their health, safety or well-being, either explicitly or implicitly (1).

According to Martino and Musri (2), the recognised forms of WPV include physical injuries, verbal abuse, racial abuse, bullying and sexual harassment. WPV can be classified into four types according to different perpetrators: Type I (criminal intent), Type II (patient/visitor), Type III (worker-on-worker) and Type IV (organisational) (3). In healthcare facilities, HCWs are most prone to Type II WPV (4). Around the globe, WPV in healthcare facilities is reportedly high and increasing (5, 6), and a similar trend is evident in Malaysia, where the reported WPV incidence was 71.3% in a public hospital (7) and 24.8% in primary care and the community-based setting (8). Overall, 70% of HCWs in Malaysia experienced verbal abuse, 33% experienced physical abuse, 25% experienced bullying, and 4% experienced sexual harassment in the workplace (4).

WPV against HCWs directly disharmonises working conditions in healthcare facilities and indirectly compromises the health of HCWs. Health service quality is compromised after every incident of violence in the workplace, causing patients to receive poor health services. Among the consequences of WPV on HCWs were depression, post-traumatic stress disorder, truancy at work, a high turnover rate at the workplace, malpractice and provision of poor health services (9, 10). According to da Silva et al. (11), WPV is a risk factor for depressive symptoms among HCWs who have experienced violence in the workplace. These workers exhibit excessive sleep behaviour and display tiredness and reduced concentration while on duty. Aside from affecting their mental health, WPV against HCWs is also a risk factor for cardiovascular disease (12) and a cause of many other adverse physical injuries (13). The literature has emphasised that whenever violence occurs in the workplace, the quality of health services will be substandard and the health of HCWs will be compromised, further undermining community health.

Several rules and regulations, such as the Occupational Safety and Health Act (OSHA) 1994, Employment Act 1955, Industrial Relations Act 1967, Minor Offences Act 1955 and Penal Code (Act 574), protect Malaysian workers from WPV. These rules and regulations allow victims to lodge reports following WPV incidents. Nevertheless, most verbal abuses, such as ridicule, innuendo and humiliation, do not constitute crimes, even if the victim is likely to sustain injuries due to these abuses. Another setback is that these laws do not ensure the safety of HCWs. Recent advances have included improved policies, procedures and guidelines on WPV, such as general guidelines for the prevention of WPV by the Department of Occupational Safety and Health (DOSH) Malaysia (2). In addition, the Ministry of Health Malaysia has launched guidelines and training modules on WPV prevention specifically for HCWs (14, 15). However, unfortunately, the prevalence of WPV against HCWs in Malaysia continues to increase despite the launch and implementation of guidelines and training modules in healthcare facilities to prevent WPV.

Progressive measures should be adopted immediately to overcome the issue of WPV in healthcare facilities. A new perspective on WPV should be sought, particularly regarding healthcare employers’ perceptions and practices towards WPV prevention. This requires a valid instrument for the assessment of healthcare employers’ perceptions and practices towards WPV prevention. A better understanding of WPV prevention can be achieved using this new instrument, as it would allow the development of the best strategies to improve current measures. However, the existing questionnaire used to assess WPV among workers relies on relatively outdated criteria based on the joint programme of the International Labour Office (ILO), World Health Organization (WHO), International Council of Nurses (ICN) and Public Services International (PSI) (1). Other existing questionnaires mostly measure a subset of WPV, such as aggression and job satisfaction, without a scoring scale (16, 17). Furthermore, none of the available questionnaires have been translated into the Malay language or adapted for local use. The aim of this study was to validate the newly developed Malay version of the Perception and Practice of Workplace Violence Prevention (PPWVP) questionnaire by addressing construct validity and instrument reliability among employers at healthcare facilities.

## Methods

### Questionnaire Development

Prior to confirmatory factor analysis (CFA), the researchers conducted a comprehensive literature review and group discussions with a panel of experts in WPV and subsequently developed the first draft of a Malay version of the PPWVP questionnaire. The steps involved in this process of questionnaire development were: i) domain identification and verification, ii) definition of the domain and components, iii) item generation, iv) formatting of the questionnaire, v) content validation, vi) face validation and vii) exploratory factor analysis (EFA). Two domains were constructed: i) the perception and ii) the practice towards WPV prevention. Each domain initially had six components and a combined total of 100 items. Two items in the practice domain were eliminated during content validation for having an item content validation index (I-CVI) below 0.78, while the remaining 98 items were kept in face validation until EFA.

Data for the EFA study were collected in the Bachok district, Kelantan, Malaysia. The gathered data were subjected to EFA analysis separately for the perception and practice domains. The EFA revealed a factor loading above 0.60 and Cronbach’s alpha above 0.71 for the perception domain and above 0.82 for the practice domain. The final draft of the Malay version of the PPWVP questionnaire comprised 62 items constructed under the domains of perception (35 items) and practice (27 items). The perception domain consisted of nine components: i) form of WPV (8 items), ii) causes of WPV (3 items), iii) impacts of WPV (3 items), iv) benefits of WPV prevention (6 items), v) barriers to WPV prevention (5 items), vi) high-strain job characteristics (3 items), vii) reaction to WPV (3 items), viii) WPV protection (2 items) and ix) WPV prevention encouragement (2 items). By contrast, the practice domain consisted of four factors: i) workplace safety (3 items), ii) implementation of WPV prevention (15 items), iii) WPV reporting (4 items) and iv) managerial role (5 items). All questions were close-ended and rated on a 5-point Likert scale ranging from 1 (strongly disagree) to 5 (strongly agree).

### Study Design and Participants

A cross-sectional study was conducted between October 2021 and November 2021 in Kota Bharu, Kelantan, Malaysia. The study involved five categories of healthcare workplaces: i) hospitals, ii) health clinics, iii) dental clinics, iv) district health offices and v) district dental offices. Healthcare facility employers who had worked at least 12 months in the current workplace, as well as representatives from any of the levels of director of the organisation, location supervisor or members of occupational safety and health committees (OSHCs) were invited to participate in the study. The estimated sample size was 333 participants. The number of participants required for each workplace category was determined using a stratified proportionate sampling formula. We gathered a list of total employers in each workplace category during the initial recruitment process and then conducted participant selection using simple random sampling. Participants who consented to participate were given an online questionnaire via email and an online messaging platform.

The current study defined the director of an organisation as an employer in charge of healthcare facilities and included hospital directors and medical officers. The location supervisor was defined as the employer in charge of the respective department in healthcare facilities and included the heads of department, senior assistant medical officers, environmental health officers and matrons. The OSHC in healthcare facilities referred to the committees consisting of safety and health officers, workers and representatives of the organisation that aimed to improve health and safety at work.

### Statistical Analysis

Data analysis in this study was performed using R software for Windows version R-4.2.1 (2022-06-23). The characteristics of the participants were analysed using descriptive analysis. The mean and standard deviation (SD) were used to describe continuous variables, whereas frequency and percentage were used to describe categorical variables.

CFA was performed using *lavaan* version 0.6–11 and *semTools* version 0.5–6 of the R packages (18, 19) to test the fit of the data in relation to the factor structure. Prior to CFA analysis, descriptive statistics for the PPWVP questionnaire were computed to measure the mean scores for every item, dimension and outcome. Assumption checking was then carried out to determine the estimator used for the analysis in this study. The scale’s dimensionality was determined using standardised factor loadings and a value of > 0.60 was accepted (20–22). An item with low factor loading was removed unless it was considered meaningful (23). The model’s goodness of fit was examined based on fit indices. Three model fit categories (absolute fit, parsimonious fit and comparative fit), their respective fit indices and the recommended cut-off values were observed (24, 25).

Assessments of model fit were carried out using the assessment item fit and model fit criteria of the standardised root mean squared residual (SRMR), root mean square error of approximation (RMSEA), comparative fit index (CFI), Tucker Lewis index (TLI) and Chi-square test (*χ^2^*). The SRMR is an absolute measure of fit and is defined as the standardised difference between the observed and predicted correlations. By contrast, RMSEA is an absolute fit index because it assesses how far the initial proposed model deviates from a perfect model. The CFI and TLI are incremental fit indices that compare the fit of a hypothesised model with that of a baseline model. The model was considered fit when the *P*-value (*χ^2^*) > 0.05, SRMR and RMSEA < 0.08, relative chi-square < 3.0, CFI and TLI > 0.90 (25–27).

The model revision was considered by removing a problematic item, changing item loading to other factors if justified on the theoretical background or combining factors if they were highly correlated. However, the absolute fit index of minimum discrepancy *χ^2^* could be ignored when the study’s sample size was greater than 200, and relative *χ^2^* was preferable for use as a fit index (28). Finally, the reliability test was determined by Raykov’s rho coefficient and a threshold equal to or greater than 0.7 was considered adequate for this study (27).

## Results

### Characteristics of Participants

Upon sampling recruitment, 333 employers of healthcare facilities were selected for the confirmatory factor analysis. The participants were obtained from five categories of healthcare facilities: i) hospitals, ii) health clinics, iii) dental clinics, iv) district health offices and v) district dental offices. These five healthcare workplaces, despite their variety, generally shared similar administration management and processing workflows.

The participants were representative of three levels of HCWs: i) directors of the organisation (6.0%), ii) location supervisors (82.6%) and iii) OSHCs (11.4%). In total, 324 were Malay (97%), 232 were women (69.7%), 297 (89.2%) were married and 230 (69.1%) had diplomas. The mean age of the participants was 45.5 years old and more than 92% had more than 10 years of working experience.

According to the workplace categories, two-thirds of the participants were from hospitals, and only one participant (0.3%) was from a district dental office (Table 1).

### Confirmatory Factor Analysis

A check of the multivariate normality of the data revealed that the data were not multivariately normal. Therefore, the robust maximum likelihood (MLR) estimation method was used in the analysis and resulted in a 13-factor structure. The CFA verified three models. Model 1 consisted of 13 factors and 62 items. All items in this model had a factor loading greater than 0.60, except for six items with low factor loadings (≤ 0.60). The six items were ‘Prevention against violence in the workplace improves the achievements of staff’ in Factor 2, ‘Violence at workplace occurs due to patient or visitor failing to control their emotions or anger’ in Factor 4, ‘I provide unfair service to the staff’ and ‘I pay less attention to the feelings of subordinates’ in Factor 12, and ‘I enjoy working with my staff’ and ‘I give support to staff who experience workplace violence’ in Factor 13 (Table 2).

Model 2 removed six underperforming items. The remaining 56 items associated with the existing 13 factors showed factor loadings of at least 0.60 and above (Table 2). However, the fit indices were inadequate. Therefore, the localised areas of misfit were examined using modification indices (MIs). This test was used to determine whether any modifications could be performed to improve the model. Overall, 17 suggested specifications had MIs > 3.84.

The best model was Model 3, which had the exact number of factors and items as Model 2 but applied modification indices. All 13 factors, comprising 56 items, showed satisfactory factor loadings greater than 0.60 (Table 2). Moreover, all the fit indices indicated adequate goodness of fit.

Table 3 shows the details of the fit indices for each model. Model 1 did not achieve the standard values for the two fit indices: CFI (0.855) and TLI (0.843). Upon removal of the six items mentioned above, Model 2 also failed to achieve standard values for two similar fit indices: CFI (0.874) and TLI (0.862). However, Model 3 showed excellent values for all fit indices, indicating that this model was the best construct for the newly developed PPWVP questionnaire.

**Figure 1:**
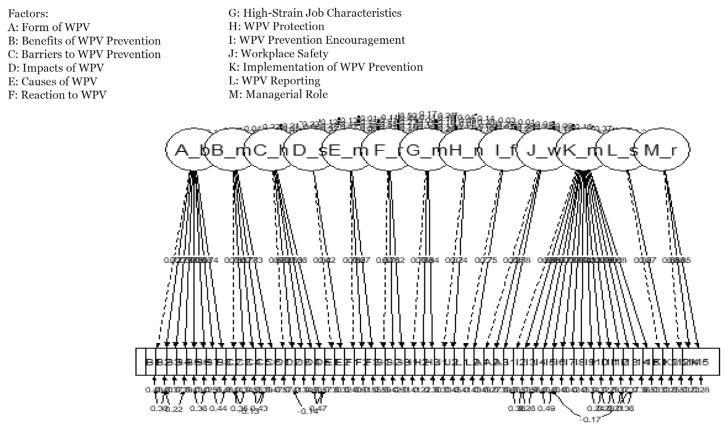
Path diagram of Model 3

Table 4 demonstrates Raykov’s rho coefficients for the three models. All factors in each model achieved Raykov’s rho coefficient values equal to or greater than 0.7. In addition, the construct reliability for all factors in Model 3 was reliable, with Raykov’s rho coefficient values ranging from 0.72 to 0.94. The path diagram of Model 3 is shown in Figure 1. The path diagram shows a standardised factor loading for Model 3, ranging from 0.621 to 0.884, which is a cut-off value > 0.6.

## Discussion

This study evaluated a psychometrically robust instrument to assess the perception and practices of WPV prevention among employers of healthcare facilities and to ensure that the instrument is culturally acceptable for use in Malaysia. The instrument is a newly developed Malay language questionnaire comprising 62 items constructed in two domains: i) perception and ii) practice. It has good content, face validation, construct and internal reliability, as determined during the early phase of the study. The current research used CFA to verify and confirm the best-fit model for the factor structures of the draft questionnaire tested with the EFA.

The CFA produced and verified three models. Model 1 showed six items (items 9, 22, 56, 57, 58 and 60) with unacceptable factor loadings and unsatisfactory fit indices values. Items with factor loadings of 0.6 and lower should be dropped from the model to improve the validity and reliability of the instrument, as these items do not contribute to measuring the construct (20–22). This is consistent with the results obtained by Mustafa et al. (29), who reported that all items in the measurement model should achieve factor loadings above 0.6 to prove that the model is unidimensional. All items should have acceptable factor loadings for their respective latent constructs to achieve unidimensionality (30). Removing the six poorly performing items in the present study might lead to distorted findings, particularly because the validation process was conducted only in a small region. Fortunately, other positive statement items with similar meanings remained in the same domain, thereby maintaining the overall objectives of the respective domains. Therefore, the intended value of the fit indices would be achieved by removing these problematic items from the model.

Model 2 was constructed by removing the six problematic items with factor loadings of 0.6 and lower. Although an acceptable factor loading was achievable in Model 2 for all the construct items, the fit index values were still unacceptable, especially for the CFI (0.87) and TLI (0.86). After applying modification indices and leaving out the six poorly performing items, Model 3 showed good factor loadings (> 0.60) for all 56 construct items and produced the best acceptable fit index result (*χ*^2^ = 2092.6, df = 1389, *P* < 0.001, CFI = 0.928, TLI = 0.920, RMSEA = 0.042 and SRMR = 0.053), as suggested by many authors (25–27).

The construct reliability for all 13 factors in Model 3, as indicated by Raykov’s rho coefficient values of 0.70 and above, was considered acceptable (31). In this study, Raykov’s rho coefficients were preferred over Cronbach’s alpha because they consider multiple factors and the differences in factor loadings among the items (32, 33). Conversely, the primary concerns with Cronbach’s alpha are the possible flaws in the assumption of unidimensionality and the consideration that all items are equally constructed with the factors (34).

This study involved five categories of healthcare facilities, namely hospitals, health clinics, dental clinics, district health offices and district dental offices in the sampling population. Although most participants were from hospitals, the heterologous study site applied in this study offered generalisability and representativeness of the data, which can be considered its prime research strength. The present study also focused on different levels of employers in healthcare facilities. This is important because WPV prevention initiatives will only be effective with support from the top, middle and low levels of management in an organisation. The initiative should be disseminated and sufficiently publicised among directors of organisations, location supervisors and OSHC members in healthcare facilities.

This study has a few limitations. One was that data were collected using online-based questionnaires via email and an online messaging platform. Many benefits of online questionnaires have been highlighted, such as their flexibility, cost-effectiveness, accessibility to participants, fewer transfer errors and no requirement for direct contact or addressing safety concerns (a particular concern during the COVID-19 pandemic) (35). Nevertheless, online questionnaires have some limitations, including a lack of verbal and direct one-way communication with participants, which may cause them to be motivationally deprived or to experience difficulty understanding the study’s intention. Further confounding factors specific to Malaysia were the stigma of being blamed and the punitive culture of the Malaysian healthcare environment. Thus, participants possibly did not appreciate particular questions, as they feared the blaming culture, even though confidentiality and anonymity were clearly stated in this study (36, 37). Another unavoidable limitation was the low response rate among the directors of organisations, possibly due to COVID-19 management time constraints.

## Conclusion

This study showed that the newly developed PPWVP questionnaire in Malay is valid, reliable and culturally acceptable. Both the perception and practice domains, together with their constructed items, were found appropriate for assessing employers’ perceptions and practices of WPV prevention at healthcare facilities. Therefore, the questionnaire can be used as an instrument for further study in local healthcare facilities and workplace settings, with some amendments to suit the particular workplace and local context.

## Figures and Tables

**Table 1:** Characteristics of participants (*n* = 333)

Variables	*n* (%)	Mean (SD)
Age (years old)		45.53 (6.21)
Gender		
Male	101 (30.3)	
Female	232 (69.7)	
Race		
Malay	324 (97.3)	
Chinese	8 (2.4)	
Others	1 (0.3)	
Marital status		
Single	12 (3.6)	
Married	297 (89.2)	
Divorced	24 (7.2)	
Educational level		
Certificate	9 (2.7)	
Diploma	230 (69.1)	
Degree	79 (23.7)	
Master	12 (3.6)	
PhD	3 (0.9)	
Work experience		
2–5 years	3 (0.9)	
5–10 years	23 (6.9)	
More than 10 years	307 (92.2)	
Ever had WPV prevention training (*Yes*)	43 (12.9)	
Communication technique (*Good*)	320 (96.1)	
Types of workplaces		
Hospitals	250 (75.1)	
Health clinics	73 (21.9)	
Dental clinics	4 (1.2)	
District health offices	5 (1.5)	
District dental offices	1 (0.3)	
Level of employer		
Director of organisation	20 (6.0)	
Location supervisor	275 (82.6)	
OSHC*	38 (11.4)	
Organisational factors for WPV prevention		
Enough funding (*Yes*)	166 (49.8)	
Policies (*Yes*)	193 (58.0)	
Safety procedure (*Yes*)	219 (65.8)	
SOP for reporting¥ (*Yes*)	223 (67.0)	

Notes:

* OSHC= Occupational Safety and Health Committee;

¥ SOP= Standard operating procedure

**Table 2:** Factor loadings of PPWVP questionnaire for three models (*n* = 333)

No	Factors/Items	Factor loading		

		Model 1	Model 2	Model 3
Factor 1 (Form of workplace violence)				
1	I believe there has been verbal intimidation in the workplace over the past year.	0.729	0.729	0.721
2	I believe there has been physical violence in the workplace over the past year.	0.786	0.786	0.772
3	I believe there has been an act of vandalism in my workplace over the past year.	0.775	0.774	0.793
4	I believe there has been attempted physical assaults on my staff over the past year.	0.778	0.778	0.778
5	I believe there has been sexual harassment in the workplace over the past year.	0.773	0.773	0.752
6	I believe there has been an act of bullying in the workplace over the past year.	0.782	0.783	0.758
7	I believe there has been racial harassment (racist) in the workplace over the past year.	0.699	0.700	0.661
8	I believe there has been an act of stalking staff at work over the past year.	0.761	0.761	0.737
Factor 2 (Benefits of workplace violence prevention)				
9	Prevention against violence in the workplace improves the achievements of staff.	0.549	Deleted	Deleted
10	Prevention of workplace violence improves the safety of staff.	0.789	0.769	0.746
11	Prevention of workplace violence can increase staff awareness of the risk of violent incidents in the workplace.	0.853	0.857	0.813
12	Prevention of workplace violence can reduce the cost of treatment that has to be borne due to the violent cases that occur.	0.707	0.718	0.671
13	Prevention of violence in the workplace can reduce the cost of compensation to be incurred as a result of the violent cases that occur.	0.769	0.776	0.780
14	Prevention of workplace violence will improve the image of the organisation.	0.703	0.700	0.728
Factor 3 (Barriers to workplace violence prevention)				
15	I have limited time to implement workplace violence prevention programmes.	0.628	0.629	0.659
16	I have financial constraints to implement workplace violence prevention programmes.	0.704	0.705	0.766
17	I have staff constraints in implementing workplace violence prevention programmes.	0.774	0.774	0.814
18	Staff working in remote areas is an obstacle to workplace violence prevention programmes.	0.733	0.773	0.630
19	Staff working shifts are an obstacle to workplace violence prevention programmes.	0.730	0.729	0.656
Factor 4 (Impacts of workplace violence)				
20	Violence in the workplace is an act that should not be accepted.	0.780	0.839	0.837
21	Violence in the workplace can injure staff and damage property in the workplace.	0.880	0.821	0.823
22	Violence in the workplace occurs due to patients or visitors failing to control their emotions or anger.	0.541	Deleted	Deleted
Factor 5 (Causes of workplace violence)				
23	Violence in the workplace is an expression of a patient’s or visitor’s feelings, much like anger or growl.	0.774	0.775	0.775
24	After committing violence in the workplace, patients or visitors feel calmer.	0.625	0.624	0.624
25	Violence in the workplace is one of the perpetrator’s methods to protect himself.	0.668	0.668	0.668
Factor 6 (Reaction to workplace violence)				
26	Violence in the workplace is a normal reaction to feelings of anger.	0.638	0.641	0.641
27	Violence in the workplace is a positive reaction caused by the anger of the patient or visitors while receiving treatment/running errands.	0.759	0.763	0.763
28	Workplace violence can help staff to improve the relationship between staff and patients.	0.626	0.621	0.621
Factor 7 (High-strain job characteristics)				
29	Workplace violence is caused by a shortage of staff working at the scene.	0.766	0.765	0.767
30	Workplace violence occurs due to an increase in the number of patients or the occurrence of overcrowding at work.	0.886	0.885	0.884
31	Workplace violence prevails due to staff working in small numbers (less than 5 people).	0.836	0.837	0.837
Factor 8 (Workplace violence protection)				
32	Workplace violence occurs due to the absence of an effective workplace violence prevention programme.	0.821	0.821	0.818
33	Workplace violence occurs due to the absence of regulation to protect staff.	0.741	0.740	0.744
Factor 9 (Workplace violence prevention encouragement)				
34	Increased treatment costs and workers’ compensation may drive the implementation of workplace violence prevention.	0.766	0.763	0.767
35	The time loss for patient care and working can encourage the implementation of workplace violence prevention.	0.753	0.756	0.752
Factor 10 (Workplace safety)				
36	I ensure electronic observation (CCTV) is provided in the workplace.	0.718	0.718	0.717
37	I ensure security guards are provided in the workplace.	0.851	0.852	0.853
38	I ensure that physical safety protection is provided in the workplace.	0.781	0.781	0.781
Factor 11 (Implementation of workplace violence prevention)				
39	I ensure workplace organisations have the power to arrest or detain individuals to be handed over to the police.	0.631	0.631	0.631
40	I ensure that workplace organisations have the power to confiscate any weapons brought into the workplace area.	0.681	0.681	0.683
41	I ensure that workplace organisations have mechanisms or methods to identify patients or visitors with a record of workplace violence.	0.667	0.667	0.664
42	I ensure that workplace organisations provide additional safety arrangements (e.g. security alarms, physical barriers at workplace stations) for staff who have been victims of workplace violence.	0.782	0.782	0.773
43	I ensure that workplace organisations have programmes or policies that prevent workplace violence.	0.775	0.776	0.776
44	I ensure that the issue of patients or visitors who are perpetrators of violence is included in the workplace violence prevention policy.	0.767	0.767	0.776
45	I ensure that workplace organisation teaches staff to report workplace violence incidents.	0.750	0.750	0.764
46	I ensure that workplace organisation deals with violent incidents that occur outside the workplace if they are related to duty and the workplace (e.g. harassment, stalking, physical injury or verbal threats).	0.737	0.736	0.744
47	I ensure that workplace organisation periodically reviews the effectiveness of workplace violence prevention programmes or policies.	0.824	0.823	0.813
48	I ensure that workplace organisation has a dedicated committee or work team that manages workplace violence prevention.	0.847	0.847	0.827
49	I ensure workplace organisation provides staff with information materials regarding the workplace violence prevention.	0.862	0.862	0.845
50	I ensure workplace organisation provides workplace violence prevention training to staff.	0.815	0.815	0.795
51	I ensure workplace organisation provides separate/additional training on domestic violence prevention if needed.	0.804	0.805	0.795
52	I ensure that staff in my organisation who have experienced any incidents of workplace violence to lodge a report, including those who have not suffered injuries.	0.786	0.786	0.798
53	I ensure that workplace violence prevention programmes or policies in the organisation improve after any workplace violence incident.	0.676	0.675	0.683
Factor 12 (Workplace violence reporting)				
54	Over the past 12 months, incidents of workplace violence at my organisation have increased.	0.814	0.817	0.818
55	Over the past 12 months, workplace violence incidents have affected staff in my organisation.	0.805	0.869	0.867
56	I provide unfair service to the staff.	0.556	Deleted	Deleted
57	I pay less attention to the feelings of subordinates.	0.570	Deleted	Deleted
Factor 13 (Managerial role)				
58	I enjoy working with my staff.	0.560	Deleted	Deleted
59	I take appropriate action when bullying occurs at work.	0.724	0.684	0.684
60	I give support to staff who experience workplace violence.	0.524	Deleted	Deleted
61	I created a safe workplace environment.	0.852	0.876	0.876
62	I accept staff opinions about workplace violence prevention programmes or policies.	0.835	0.848	0.847

**Table 3:** Model fit indices of the PPWVP questionnaire for three models (*n* = 333)

Model	CFA index standard	Model 1	Model 2	Model 3
*χ^2^* (df)	–	3295.4 (1751)	2623.6 (1406)	2092.6 (1389)
*P*-value	> 0.05	< 0.001	< 0.001	< 0.001
*χ^2^*/df	< 3.0	1.9	1.9	1.5
RMSEA (95% CI)	< 0.08	0.055 (0.052, 0.058)	0.055 (0.051, 0.058)	0.042 (0.038, 0.045)
SRMR	< 0.08	0.060	0.055	0.053
CFI	> 0.90	0.855	0.874	0.928
TLI	> 0.90	0.843	0.862	0.920

Notes: *χ^2^* = Chi-square; df = degree of freedom; RMSEA = root mean square error of approximation; SRMR = standardised root mean squared residual; CFI = comparative fit index; TLI = Tucker-Lewis index; CI = confidence interval

**Table 4:** Raykov’s rho of the PPWVP questionnaire (*n* = 333)

Factors	Raykov’s rho		

	Model 1	Model 2	Model 3
Form of workplace violence	0.92	0.92	0.88
Benefits of workplace violence prevention	0.86	0.87	0.83
Barriers to workplace violence prevention	0.84	0.84	0.81
Impacts of workplace violence	0.79	0.82	0.82
Causes of workplace violence	0.74	0.74	0.74
Reaction to workplace violence	0.72	0.72	0.72
High-strain job characteristics	0.87	0.87	0.87
Workplace violence protection	0.76	0.76	0.76
Workplace violence prevention encouragement	0.73	0.73	0.73
Workplace safety	0.82	0.82	0.82
Implementation of workplace violence prevention	0.95	0.95	0.94
Workplace violence reporting	0.79	0.83	0.83
Managerial role	0.81	0.84	0.84
